# Novel Method for Pairing Wood Samples in Choice Tests

**DOI:** 10.1371/journal.pone.0088835

**Published:** 2014-02-14

**Authors:** Sebastian Oberst, Theodore A. Evans, Joseph C. S. Lai

**Affiliations:** 1 Acoustics & Vibration Unit, School of Engineering and Information Technology, The University of New South Wales, Canberra, Australia; 2 Department of Biological Sciences, National University of Singapore, Singapore; The Pennsylvania State University, United States of America

## Abstract

Choice tests are a standard method to determine preferences in bio-assays, e.g. for food types and food additives such as bait attractants and toxicants. Choice between food additives can be determined only when the food substrate is sufficiently homogeneous. This is difficult to achieve for wood eating organisms as wood is a highly variable biological material, even within a tree species due to the age of the tree (e.g. sapwood vs. heartwood), and components therein (sugar, starch, cellulose and lignin). The current practice to minimise variation is to use wood from the same tree, yet the variation can still be large and the quantity of wood from one tree may be insufficient. We used wood samples of identical volume from multiple sources, measured three physical properties (dry weight, moisture absorption and reflected light intensity), then ranked and clustered the samples using fuzzy *c*-means clustering. A reverse analysis of the clustered samples found a high correlation between their physical properties and their source of origin. This suggested approach allows a quantifiable, consistent, repeatable, simple and quick method to maximize control over similarity of wood used in choice tests.

## Introduction

Choice-tests are perhaps the most common experimental method used to determine preferences of insects, especially for food. A simple ISI search for papers on ‘choice tests’ to determine food preferences of insects found around 600 papers from the past decade. One common use of food choice tests is to determine food additives (e.g. bait substrates, attractants, toxicants) for pest control applications, e.g. for cockroaches [1], [2], moths [3], ants [4], [5] and termites [6].

Choice-tests for food additives in artificial foods are straightforward as the base food matrix is identical across the choices under test. Those for food additives in natural foods are more problematic as the natural foods are often highly variable, thus the palatability of the base matrix may confound the effect of the food additive. Wood is such a variable food, not just between tree species but within any one tree species, owing to the age of the fibre, the horizontal (sapwood versus heartwood) and vertical position within the stem as well as ecological (e.g. growth site and conditions: natural vs. plantation, growth rate) and functional (e.g. stems vs. branches, reaction wood formed in leaning stems and branches and juvenile vs. mature growth) variations [7], [8], [9], [10]. Such variation has been demonstrated to affect termite consumption of wood, especially due to the age of a tree [11], [12], [13]. Other factors identified include moisture [14], [15] or previous termite attack [16].

Hence the interpretation of wood loss in choice experiments [17], [18], [19], [20], [21] may not be straight forward because wood consumption may have differed due to inter-sample palatability as well. Attempts to reduce this natural variation have used sequentially cut wood [22], [23], [24]; however, there is variation in the wood quality within logs cut from the same tree [25], [26], owing to growth increment variation, for example. Complicating this limitation is unknown provenance, when wood for laboratory experiments on termites may have been sourced from retailers. This is most problematic for large experiments that require more wood samples than one retail sourced cut length of timber can provide.

A simple, consistent, repeatable method to characterise wood using easy to measure physical properties would reduce the variability within samples for choice tests, and thus increase confidence in results. The aim of this paper is to test the similarity of wood samples cut sequentially from different *Pinus radiata* sources, by applying *fuzzy c-means clustering* [27], [28], to three simple measurements of physical properties: dry weight, moisture absorption and reflected light intensity. Fuzziness in the algorithm allows selecting wood pieces from different clusters which accounts therefore for the uncertainty of material properties that are not measured.

## Materials and Methods

### Physical Properties of Wood

The wood used was plantation grown *P. radiata*, cut as veneer into sheets (ca. 1250 mm×25 mm×1 mm), from a retailer; thus the source trees were unknown. Veneer discs (60 mm 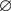) were punched from each sheet and were given a unique identification code, which included the original sheet from which the samples were cut (hence ‘sheet membership’). Only undamaged veneer discs without knotholes and obvious fungal attack were chosen.

Two sets of veneer discs were prepared, for which the veneer was most likely from different trees or even different geographical locations. In any case the two sets represented a variation in sets for the statistical analysis: for the Small Set, *N* = 505 discs were cut from 10 sheets; and for the Large Set, *N* = 1417 discs were cut from 22 sheets. For both sets three physical properties were measured. (1) The dry weight was recorded after discs were held for 7 hrs at 105°C in a drying oven (4 hrs for weight to be stabilised). (2) The moisture absorption was recorded as a percentage of the dry weight, with the oven dried veneer discs kept for 4 days at 28°C and 80% RH, calculated as (wet weight – dry weight)/dry weight×100. All weights were measured to four significant figures (AEA 250 g, Adam Equipment Co Ltd, Milton Keynes, UK). (3) The brightness was recorded as the mode-skewness of the reflected light (pixel intensity (*I*) distribution) from digital photographs (1660×1200 pixels) taken with a tripod mounted camera (µ tough 12 MP, Olympus). Flash was not used to avoid 50 Hz flickering; instead constant lighting was provided by six arrays of white LED packages (ea. 25×3**mm LEDs, avg. *I* = 5**cd (ea.), peak wavelength at 465 nm). The mode skewness of the reflected light calculated as (mean - mode)/standard deviation [29] was used as an estimate of the ratio of early- and late wood in the sapwood to heartwood, as the former is pale (negative mode skewness) and the latter is dark.

Photographs of 2 MP were found sufficient as distributions of photographs with higher resolutions (3 or 5 MP) did not show different results in distribution parameters but increased the computational load from 8 hrs to 1 and 3 days respectively. Images were processed from 16 bit colour depth to 8 bit (gray) and a cut-off pixel intensity of 60 was selected from plotting sorted veneer intensities over pixels for all discs; pixels with *I* <60 represented black background and were discarded. The intensity distribution and its mode-skewness were calculated for each veneer disc. Signal processing and optimisation was performed in Matlab R2012 and the statistical analyses were performed with RStudio 0.97.332 and R x64.2.13.1.

### Determination of Similarity

The analysis process had three steps. Step 1 calculated fuzzy *c*-means derived from the measured physical properties only; in other words the origin of the veneer discs was not known. Step 2 used the known origin of the veneer discs, here termed ‘sheet membership’, to search for neighbouring veneer discs. Step 3 from fuzzy *c*-means derived clusters, uniform distributions was assigned to sheet membership in each cluster found in the experimental data with the same length (referred to as ‘experimental uniform data’). This ‘experimental uniform data’ was benchmarked against a hypergeometric distribution, thus allowing the effectiveness of fuzzy *c*-means clustering to find similar veneer discs to be assessed. The hypergeometric distribution describes a process equivalent to drawing balls from an urn but without replacement [30]. For this purpose the clustered groups were subsequently degraded in order to benchmark the clustering algorithm by comparing the effect of having completely randomised draws and the effect of the size of the population on the sorting quality of the fuzzy *c*-means clustering. Benchmarks with respect to neighbourhood size and the significance of the performance of the clustering algorithm with respect to medians calculated were discussed.

### Clustering

A statistical cluster analysis used optimised subsets to group elements. Fuzziness was implemented to take the uncertainty into account, e.g. by assigning one veneer disc to several clusters. Fuzziness also implies that the method approximates “non-crisp” (not optimal) values [28].

The fuzzy *c*-means clustering algorithm [28] was implemented in Matlab R2012, and based on minimising the cost function [27], [28]

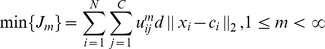
(1)with *m*>1 being the fuzzifier (default *m* = 2), *x_i_* the *i*th vector of dimension *d*, *u_ij_* the element of the partition matrix of the veneer disc *x_i_* in a cluster *j* describing the membership grade, and with ||.||_2_ as the Euclidean distance between the vector (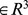) and its cluster centre to be evaluated (*c_j_* is the number of clusters). Fuzzy *c-*means clustering iteratively approximated the mean centroid of each cluster, from which a membership value for each veneer disc was calculated based on the strength of the association between the element and the centroid. A list of veneer discs from each cluster was sorted in descending membership order; the closer the membership to 1, the more the veneer disc belongs to the cluster. In order to partition each cluster in one set with unique membership and one fuzzy set, a second distance function was applied to sub-cluster veneer discs in subsets of lower membership (*u_ij_ <*0.5) into a fuzzy region.

### Sorting Quality and Statistics

The fuzzy *c*-means algorithm gives clusters which are optimised according to their material properties and are characterised by (1) a sequence of veneer discs, (2) sheet memberships of veneer discs and (3) number of veneer discs in the cluster (referred to as cluster length). The accuracy of the assignment of veneer discs into similar groups using fuzzy *c*-means clustering process was tested by comparing these groups with sheet membership.

Veneer discs were mapped using their descending membership grade order in each cluster list, which created a spiral in a 3-dimensional veneer disc property space, starting from a region with unique cluster membership, and ending in a fuzzy region. The clustering can therefore be seen as a mapping from the 3-dimensional space onto a 1-dimensional subspace of membership grades.
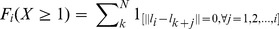
(2)


Equation (2) gives a Euclidean distance as an argument of the indicator (or characteristic) function 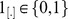 [29]. The indicator function searched for neighbouring veneer discs from the same sheet, which were then given a value of one, all others assigned zero. The percentage of successful neighbour searches relative to the population’s vector length was calculated for each neighbourhood size. From the centroid, the optimisation procedure moved iteratively outwards, such that the neighbourhood size was successively increased, by checking first direct neighbours, then second neighbours and so on. The percentage of neighbours was subsequently interpreted as the probability of encountering at least one neighbour of the same sheet 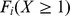 for discrete neighbourhood widths 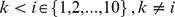. This was compared to 8 averages of bootstrapped uniform data of clusters of the same length and all sheet memberships as the experimental data and a hypergeomeric distribution (*n* = 8000 draws).

In order to find out the influence of the cluster length obtained by fuzzy *c*-means clustering, the probabilities to encounter neighbours were benchmarked against bootstrapped uniform distributions of sheet membership, first for clusters with the same length as the experimental data and then for clusters with identical length.

Thus the order of sheet membership was randomised so that the membership matrix (i.e. the list) for each cluster was not sorted. This corresponded to theoretical sets of identical weighted values, hence reduced the dependency of the measured wood properties, which led to the degradation of results obtained by the optimisation algorithm. For clusters with identical lengths, the bootstrapped averaged samples (8 repeats) for the Small Set had a total number of 360 veneer disc samples per sheet (10 sheets with *n* = 36 veneer discs each) and for the Large Set 506 veneer discs (22 sheets, *n* = 23 veneer discs each).

Distribution (Lilliefors-test) and significance tests (Student’s *t*- test or Fisher’s one-way ANOVA) were used to determine whether differences in the probability in encountering at least one direct neighbour were statistically significant for the ‘experimental uniform data’.

## Results

### Physical Properties of Wood

**Figure 1:**
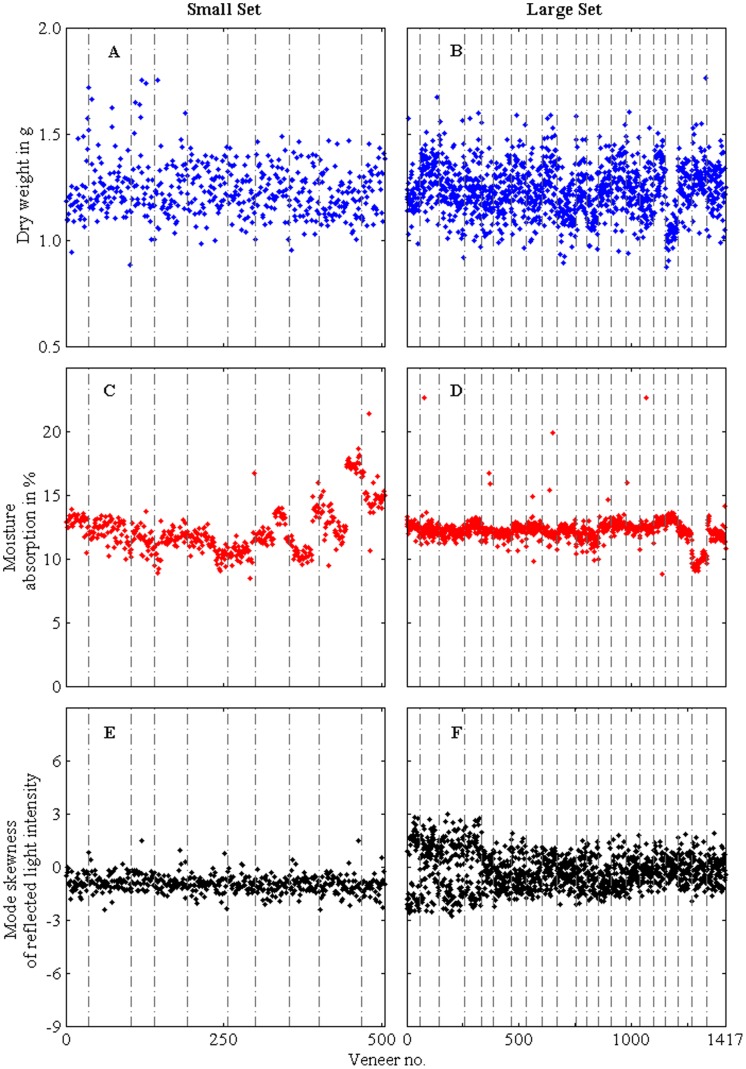
Veneer disc properties of dry weight (A & B), moisture absorption (C & D) and mode-skewness of pixel intensity (E & F) for the Small Set (*N* = 505) and the Large Set (*N* = 1417); vertical lines indicate different source sheets.

The physical properties of veneer discs are plotted in Figure 1, with vertical lines separating veneer discs from different sheets The average dry weight was 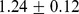 g (the Small Set) and 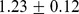 (the Large Set); the average moisture absorption was 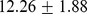
% (the Small Set) and 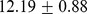
% (the Large Set); and the average mode-skewness was 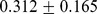 (the Small Set) and 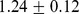 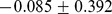 (the Large Set). A randomly drawn two sample homoscedastic *t*-test showed that for weight and moisture absorption, the hypothesis that the property’s means are equal could not be rejected (*df* = 1750, *p* = 0.990 and *p* = 0.885), while for the skewness this could not be assumed (*df* = 1750, *p* = 0). The confidence intervals (*ci*) on the difference between the means did contain zero for dry weight and moisture (*ci* = [−0.0044, 0.0042] and *ci* = [−0.0059, 0.0038]) but not for the mode skewness (*ci* = [0.2269,0.2426]).

### Clustering

**Figure 2:**
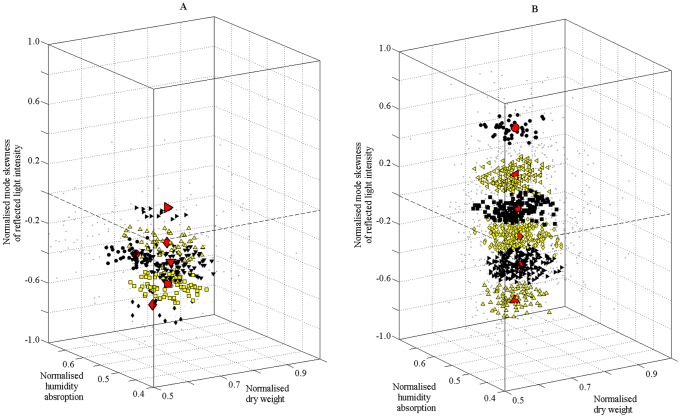
The six clusters formed of dry weight, moisture absorption and mode skewness obtained by fuzzy *c*-means optimisation algorithm; A Small Set and B Large Set. Large red markers are the cluster centres; medium black and yellow dots are members of only one cluster; small grey dots are members of more than one cluster.

For the Small Set (Figure 2**A**) all cluster centres were in regions of negative mode-skewness, which indicated that most of the wood veneer had more bright (early wood) than dark (late wood) regions [31]. The cost function was minimised to 3.5 after 15 iterations. For the Large Set (Figure 2**B**) the inner parts of the six clusters (unique membership) are depicted together with their centres, surrounded by elements with weaker membership which could belong to more than one cluster (in gray). The cost function converged after 15 iterations to a lower value, about 2.9. Therefore, it was easier to distinguish clusters for data from the Large Set than for the Small Set, where differences between measurements were larger with also a smaller value of the cost function. For the Large Set four of the six centres were in regions of negative skewness, indicating that most of the wood veneer had more bright (early wood) than dark (late wood) regions.

### Sorting Quality and Statistics

**Figure 3:**
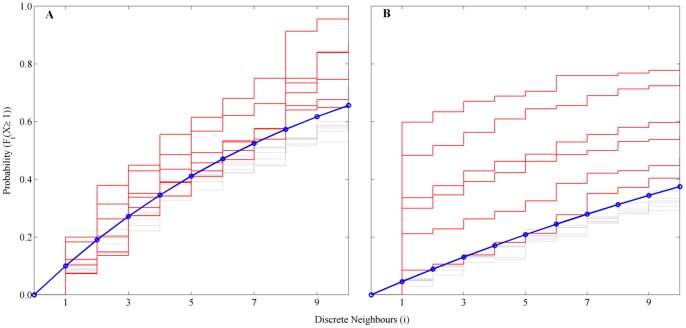
Cumulative probabilities of having at least one discrete neighbour in a neighbourhood *i* = 1…10; A Small Set and B Large Set. Solid red lines are the original distribution optimised with fuzzy *c*-clustering; solid blue line is the average (*n* = 8) of the six bootstrapped clusters (same length as the original cluster length but bootstrapped over a uniform distribution of sheet membership of all sheets); dotted grey lines are the hypergeometric (analytical) cumulative density functions.

In Figure 3, in terms of the values of cumulative probabilities to encounter at least one neighbour in an *i*-neigbourhood (*i = 1…10*), the sorting quality of the clustered data (solid red line) was benchmarked against a uniform distribution of sheet membership (averaged *n* = 8 times) with randomised elements in a cluster of length the same as the experimental data (blue line) and a hypergeometric distribution over neighbourhood size (dashed lines). The probabilities of the uniform distribution were below the probabilities obtained of the experimental data and approximated reasonably well the probabilities of the hypergeometric distribution. For comparison, the probability of the uniform distribution with one direct neighbour was 10% and 4.5%, for the Small Set and the Large Set respectively. The estimated probabilities of the experimental data having one direct neighbour (*i* = 1) in the six clusters were 7.3%, 7.4%, 10.3%, 12.3%, 18.3% and 20% for the Small Set, and 8.5%, 21.1%, 30%, 33.6%, 48.3% and 59.8% for the Large Set (averaged over 8 bootstrapped results) respectively. For the Small Set, the difference between the probabilities of the hypergeometric distribution and that of the experimental data was always smaller than that for the Large Set.

**Figure 4:**
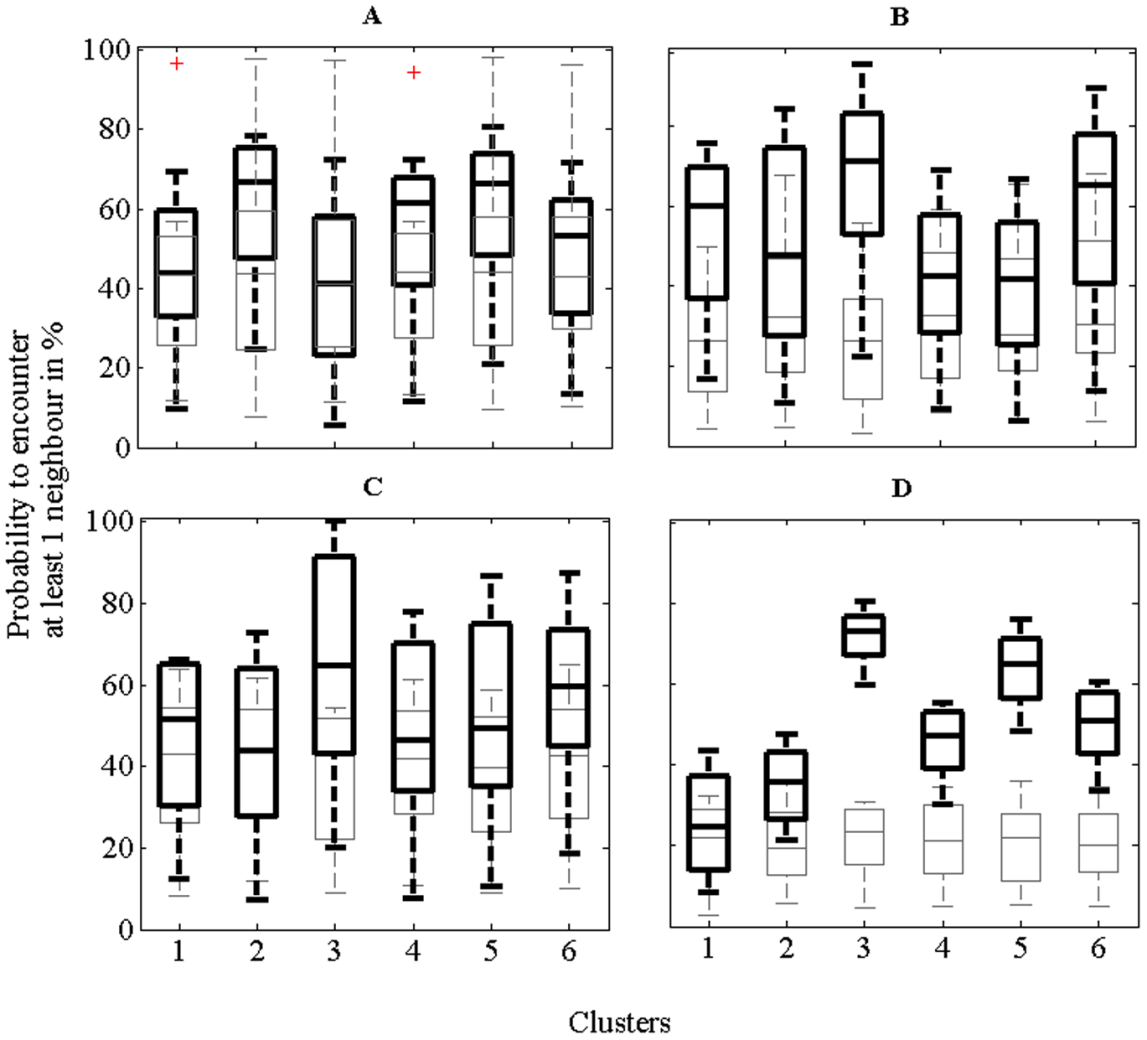
Box plots for the six clusters showing the cumulative probability to obtain at least one same sheet with respect to the neighbourhood function;+are outliers. Bootstrapped (*n* = 8) ‘uniform experimental data’ based on the original distribution (thick black lines) benchmarked against uniform distributions (*n* = 8) with the length of each cluster the same as the experimental data: **A** Small Set (*N* = 505) and **B** Large Set (*N* = 1417); and with identical cluster length for each set: **C** Small Set (*N = *360) and **D** Large Set (*N = *506).

The *H_0_* hypothesis that the distributions of the median probabilities of the experimental data as plotted in Figures 4**A and 4B** belonged to a family of normal distributions (Lilliefors test) could not be rejected at a 0.1%-level. For the experimental data within a cluster in Figures 4**A and B** for the Small Set and the Large Set respectively, a uniform distribution (uniform experimental data) was assigned to the sheet membership obtained by clustering the measured data (thick black lines). For the ‘experimental uniform data’ depicted in Figures 4**A and B** for the Small Set and Large Set respectively for cluster length as optimised by the fuzzy *c*-means algorithm, a uniform distribution over sheet membership (which included all sheets of the set) was assigned as benchmark (thin grey lines). A one-way ANOVA indicated that the median probabilities of the ‘uniform experimental data’ were significantly different from those of the benchmark distributions for the Small Set (*F = *9.55, *df* = 18, *p* = 0.0114), and for the Large Set (*F = *14.31, *df* = 18, *p = *0.0035). For the Small Set, the median cumulative probability of the benchmark distribution (ranging from 41% to 44%) was always lower than that of the ‘uniform experimental data’ (ranging from 41% to 66.7% with the fuzzy *c*-means clustering algorithm) as seen from Figure 4**A**. This was true for the Large Set also with the median cumulative probability being always higher in all clusters than that of the benchmark distribution (Figure 4**B**).

Next the effect of the length of a cluster was tested by intentionally reducing the populations of the six clusters to the minimum cluster size minus five samples, i.e. 360 for the Small Set and 504 for the Large Set. The distribution of the sheet membership was uniform within each cluster. This statistical model is complementary to the average of the six bootstrapped clusters with a uniform sheet membership distribution plotted in Figure 3 (blue line). While in Figure 3 and Figures 4**A and B**, the length of each cluster was different, the length of each cluster in Figures 4**C and D** was made identical but the sheet membership reflects the sorting quality of the clustering algorithm. Ten populations of sheet memberships of the original clusters were drawn and replaced. The results for the experimental data (thick black lines) and for the benchmark distributions (thin grey lines) with the same cluster length are depicted in Figure 4**C and D**.

The estimated probabilities of the ‘uniform experimental data’ having at least one direct neighbour in the six clusters were now 54%, 42%, 64%, 48%, 52% and 60% for the Small Set in Figure 4**C**, and 22%, 39, 77%, 54%, 62% and 51% for the Large Set (averaged over 10 bootstrapped results) in Figure 4**D**. While for the Small Set, the median cumulative probability to have at least one neighbour in Figure 4**C** decreased for clusters 2, 4 and 5 and increased for clusters 1, 3 and 6 compared to Figure 4**A**, the 25 and 75 percentiles (range measure) increased for all clusters. For clusters 1,2 and 6 of the Large Set, the median cumulative probability of having at least one neighbour in Figure 4**D** decreased compared with Figure 4**B** but increased for clusters 3, 4 and 5. However, its range measured by the 25 and 75 percentile decreased substantially which is different to the Small Set. In comparison, for both the Small Set and the Large Set depicted in Figure 4**C and D** the probability to obtain at least one neighbour using a benchmark distribution was always lower.

## Discussion

Although the difference between the Small and Large Set for the mode skewness of the reflected light intensity was statistically significant, it was not true for the other two properties (dry weight and moisture absorption) and the assignment to the original sheets of veneer with respect to one set was not obvious to visual inspection (c.f. Figure 1). However, the clustering algorithm was able to place the veneer discs into clusters that match their original source veneer sheets by using just the three measurements of physical properties. Within clusters, each veneer disc could be matched to its nearest neighbour by a threshold probability for the closest pair to be used in choice experiments.

The results for the Large Set indicated a higher probability to encounter at least one neighbour and hence it was more likely for the wood of the same sheet to have the same membership grade in the clusters obtained by fuzzy *c*-means clustering (Figure 3). The Small Set was more difficult to cluster, as reflected by a 20.6% higher cost function, owing to the smaller sample size and little variation in the mode skewness of the reflected light intensity (c.f. Figure 2). However, the clustering algorithm is robust enough to enable clusters to be formed even under these limitations.

Figures 4**A and B** show that for both the Small and Large Sets, even when the sheet membership of each cluster was randomized with a uniform distribution of sheet membership (‘uniform experimental data’), there was a high correlation between the veneer discs and the sheets from which they were cut. This observation was also true even when the length of each cluster was made identical (‘balanced’ analysis) within the Small Set and the Large Set (Figures 4**C and D**). As only three simple measured physical properties were used here, the power of the clustering algorithm could be improved to enable more precise matches by including additional physical properties such as damping, resonant frequencies.

The standard approach of pairing matched wood samples contains “uncontrolled variations” which may significantly influence the outcomes of choice experiments. Results here show that when the wood source or species is unknown, the fuzzy *c*-means clustering will give higher confidence in pairing matched wood samples. When the wood source and species is known, the same process will give a more precise match. The fuzzy *c*-means clustering process allows a greater potential to control the least controlled variable in bio-assay type experiments so that results can be attributed more confidently to the applied treatments under test, such as food additives.
